# Preparation of Polytetrafluoroethylene Superhydrophobic Materials by Femtosecond Laser Processing Technology

**DOI:** 10.3390/polym16010043

**Published:** 2023-12-21

**Authors:** Shuangquan Zhou, Yayue Hu, Yao Huang, Hong Xu, Daming Wu, Dong Wu, Xiaolong Gao

**Affiliations:** 1 College of Mechanical and Electrical Engineering, Beijing University of Chemical Technology, Beijing 100029, China; zsq20210620@163.com (S.Z.); yayuehu2022@163.com (Y.H.); hy06@163.com (Y.H.); xuhong@mail.buct.edu.cn (H.X.); wudaming@vip.163.com (D.W.); 2 State Key Laboratory of Organic-Inorganic Composites, Beijing University of Chemical Technology, Beijing 100029, China; 3 College of Engineering Science, University of Science and Technology of China, Hefei 230027, China

**Keywords:** polytetrafluoroethylene, superhydrophobicity, femtosecond laser processing, micro- and nano-fabrication

## Abstract

In recent years, superhydrophobic surfaces have attracted significant attention due to their promising applications, especially in ice prevention, reduction in air resistance, and self-cleaning. This study utilizes femtosecond laser processing technology to prepare different surface microstructures on polytetrafluoroethylene (PTFE) surfaces. Through experiments, it investigates the relationship between the solid–liquid contact ratio and surface hydrophobicity. The shape of water droplets on different microstructure surfaces is simulated using ANSYS, and the relationship between surface microstructures and hydrophobicity is explored in the theoretical model. A superhydrophobic surface with a contact angle of up to 166° was obtained by machining grooves with different spacings in polytetrafluoroethylene sheets with femtosecond laser technology. Due to the micro- and nanostructures on the surface, the oleophobicity of the processed oleophilic PTFE surface is enhanced.

## 1. Introduction

Various natural surfaces exhibit superhydrophobic properties, such as the lotus leaf surface [1], rice leaf surface [2], and butterfly wing surface [3], which exhibit superhydrophobic water. These intriguing surfaces have attracted the interest of researchers due to their potential applications in self-cleaning [4,5,6], anti-icing [7,8,9], oil–water separation [10,11,12], and directed water transfer [13]. For instance, the surface of lotus leaves is coated with a layer of low surface energy waxes and numerous micro- and nanoscale papillae structures [14], contributing to their superhydrophobicity. It has been observed that both surface micro- and nanostructures and low surface energy play significant roles in determining superhydrophobicity.

PTFE (polytetrafluoroethylene) is a material with low surface energy that has a non-polar, high contact angle, a high crystalline and ordered structure, chemical stability and corrosion resistance, high temperature stability, and other characteristics. These characteristics make it repulsive to polar materials. Water has a large hydrophobic angle because its molecules have a chain structure arranged in an orderly crystalline structure, which makes the surface of PTFE smooth. It also has excellent corrosion resistance, making it widely used in the chemical industry and chemical laboratories [15,16]. Therefore, it is widely used in many fields, including non-stick coating, non-stick pans, sealing material, the chemical industry, the electronics industry, and medical and food processing fields [17].

To fabricate superhydrophobic surfaces, researchers primarily employ techniques such as the template method [18,19,20], electrostatic spraying [21,22,23], chemical deposition [24,25,26], and electrochemical deposition [27,28,29].

Zhiqing Yuan et al., inspired by the self-cleaning superhydrophobic taro leaf, obtained a polystyrene (PS) film with a superhydrophobic surface using a natural taro leaf as a template. The water contact angle and the sliding angle of the superhydrophobic PS surface were 158° ± 1.6° and 3°, respectively [30]. Zhu et al. employed an electrostatic coating method to create a superhydrophobic character with a hydrophobic angle of 164° and an oleophobic inclination of 148°. They utilized an electrostatic spraying system to apply polysulfide powder, fluorinated ethylene propylene, and multiwall carbon nanotubes onto a solid surface treated with epoxy resin. After the coating hardened, a superhydrophobic surface was obtained [21].

However, several commonly used processing methods have their limitations. For example, the template method produces less stable surface micro- and nanostructures, and the microstructures are susceptible to damage. Electrostatic spraying may cause surface inhomogeneities. Chemical deposition is time-consuming and produces environmentally harmful compounds. Electrochemical deposition is limited to metal substrates and cannot be used for insulating materials.

Femtosecond lasers offer distinct advantages in micro/nanostructure machining due to their ultra-short pulse duration, high peak power, and minimal thermal impact [31]. For instance, precise control over the microscale morphology of material surfaces can be achieved through femtosecond laser processing by adjusting parameters such as scanning speed, pulse frequency, energy, and pattern. This enables the creation of characters with diverse morphological features [32]. The narrow pulse width of femtosecond lasers enables high peak power even at lower pulse energies, leading to the instant removal of material from the surface upon focusing. Thermal diffusion and melting times are typically tens to hundreds of picoseconds for different materials. In micro/nanostructured materials using femtosecond lasers, the pulse duration of the femtosecond laser is shorter than the time required for thermal diffusion. As a result, the deposition rate of laser energy on the surface exceeds the thermal diffusion rate, reducing the thermal effects during the ablation of the material and minimizing the melting of the surrounding material [33].

Femtosecond laser fabrication of superhydrophobic surfaces is favored by researchers. Yan et al. utilized high-power picosecond lasers and high-speed scanning mirrors to create micro-scale pit structures on molded steel. These pit structures were then replicated on silicone rubber surfaces, resulting in superhydrophobic surfaces. They employed different methods, including single-point pulses and line scans, to form regular papillary structures on the open surfaces and closed features, respectively. This approach led to contact angles of 151.5° and 150.3° for the superhydrophobic surfaces [34]. Kai Yin and colleagues have developed a porous LAP film that can switch between superhydrophilicity and superhydrophobicity via alcohol prewetting or drying using femtosecond laser line-by-line scanning technology. The film’s optical transmittance can also be controlled, enabling it to switch between transparent and opaque states [35]. R. De Palo et al. studied the generation of laser-induced periodic surface structure (LIPSS) on a quartz surface using a linearly polarized femtosecond laser pulse and compared its wettability with that of the original quartz. The contact angle measurements showed that the induced quartz had a superhydrophilic property with a contact angle of 7.6°, while the original quartz had a simple hydrophilic property with a contact angle of 41.2° [36]. Annalisa Volpe and Liu, M., introduced three methods of surface microstructure by laser machining, namely laser-induced periodic surface structure (LIPSS), direct laser interference pattern (DLIP), and direct laser writing (DLW), to obtain superhydrophobic surfaces with anti-icing behavior. Since the three laser structuring methods compensate for one another in either processing speed or structure scale, combined laser surface patterning is more advantageous in large-area processing, particularly bioinspired superhydrophobic surfaces [37,38]. The latest research shows that laser processing can achieve both hydrophobic and hydrophilic surfaces.

This article uses femtosecond laser processing technology to create surfaces of different sizes and structures on the surface of PTFE films, resulting in surfaces with different contact angles. Through software simulation calculation, the hydrophobic angle of water on different microstructure surfaces is obtained through the establishment of a simulation model based on the liquid hydrophobic theory and then combined with experiments to verify the hydrophobic angle simulation model. The simulation model is provided for the later study of the influence of microstructure on hydrophobic angle.

## 2. Experimental Section

### 2.1. Material

In this study, a 30 mm × 30 mm × 1 mm sheet of polytetrafluoroethylene (PTFE) was obtained from Shenzhen Hexoxide Plastic Hardware Company and used as the substrate for processing. Polytetrafluoroethylene (PTFE) has excellent hydrophobicity due to its unique chemical composition and structural characteristics. Its extremely low surface energy and high contact angle make it difficult for liquids to wet the surface of PTFE, which is a good material for preparing superhydrophobic surfaces.

The surface’s structure and dimensions were observed and measured using the OLS4100 laser confocal microscope manufactured by Japan’s Olympus Company and the DM4 P polarized light microscope from Germany’s Leica Company. Additionally, the hydrophobic angle of the surface was determined using the SL200KB optical contact angle/interfacial tensiometer provided by American Kono Industries Co., Ltd. (Seattle, WA, USA).

### 2.2. Characterization

**Figure 1:**
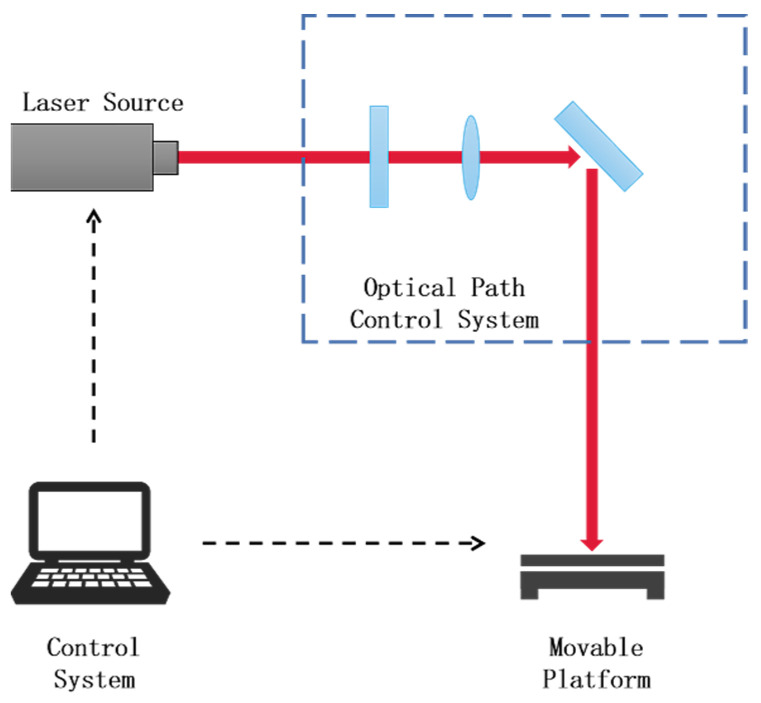
Femtosecond laser processing systems.

A Tangor femtosecond laser from France’s Amplitude was used to process the PTFE sheet. The femtosecond laser processing system comprises a control system, a laser source, an optical path control system, and a movable platform, as shown in Figure 1. The control system regulates the processing parameters of the femtosecond laser, such as pulse width, repetition rate, and scanning speed. It also controls the movement of the movable platform. The laser source emits amplified femtosecond laser light. The optical path control system consists of lenses, reflectors, and other optical instruments that guide the femtosecond laser toward the surface of the processed object. The movable platform serves as the base on which the instrument is positioned. Moving and scanning the femtosecond lasers across the object’s surface introduces potential interference and diminishes the precision of the process. To circumvent errors caused by moving the laser beam, laser scanning is often achieved by controlling the movement of the movable platform [35,39,40].

Figure 1 is the schematic diagram of the laser fabrication system. The femtosecond laser source used in our study was a Ti:Sapphire laser system with a pulse duration of 500 fs, a wavelength of 800 nm, and a repetition rate of 500 kHz. The laser beam was focused onto the PTFE surface using a plano–convex lens with a 50 mm focal length, resulting in a beam width of approximately 7 µm. The M2 factor, which characterizes the beam quality, was measured to be 1.2 using a knife-edge technique.

### 2.3. Processing

Experimental materials: PTFE sheet produced by Shenzhen Hexoxide Plastic Hardware Company and anhydrous ethanol. After ten minutes of ultrasonically cleaning the PTFE sheet in anhydrous ethanol, the sheet was dried in an oven. The processing parameters for the femtosecond laser were configured as follows: Table 1: femtosecond laser processing parameters [41]. These parameters, specifically the laser energy density, scanning speed, repetition rate, pulse width, scan spacing, and scan path, are mainly obtained from the literature references and actual processing and debugging.

After drying, the PTFE sheet was positioned on the sample table and subsequent operations were carried out using the femtosecond laser control system. After the treatment, the sample underwent a cleaning and drying process again.

### 2.4. Theoretical Analysis

**Figure 2:**
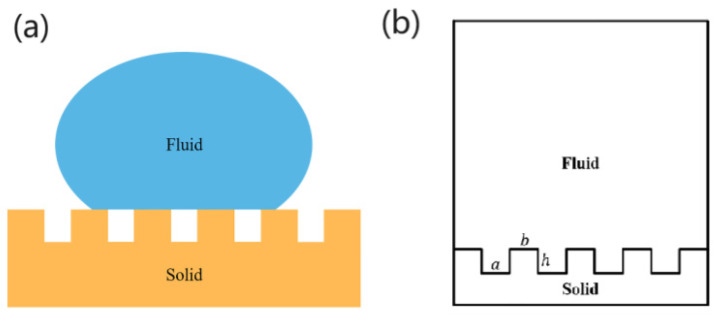
(**a**) Diagram of the Cassie model and (**b**) schematic diagram of the two-dimensional model.

The Cassie model proposed by Cassie suggests that droplets on a rough solid surface are not fully immersed in the surface structure but are instead supported by bubbles within the microstructure, as shown in Figure 2a. Cassie proposed that the contact between the droplet and the solid surface represents a three-phase contact involving solid–liquid–gas interfaces. From this, he derived the corresponding contact angle formula [42].

(1) $$\cos\theta =f_{s}\cos\theta_{s}+f_{a}\cos\theta_{a}$$

where θ is the actual contact angle, $\theta_{s}$ is the contact angle of the droplet on a smooth surface, i.e., is the intrinsic contact angle, $\theta_{a}$ is the contact angle of the droplet with the gas, $f_{s}$ is the contact area, $r_{1}\;$ is the roughness of the water droplet with the solid on the solid–liquid contact surface as a percentage of the projected area of the solid–liquid contact surface, then $f_{a}$ is the contact area of the water droplet with the gas on the solid–liquid contact surface as a percentage of the projected area of the solid–liquid contact surface. When the gas is air and the droplet is water, $\theta_{a}=180{}^{\circ}$, $f_{s}+f_{a}=1$. The Cassie model can be translated into the following equation [43].

(2) $$\cos\theta =r_{1}\cdot f_{s}\cos\theta_{s}-\left(1-f_{s}\right)$$

The solid surface of the same material, $\cos\theta_{s}\;$ is the same, so here it will be set as a constant. We can determine that as the value $f_{s}$ increases, the value of cosθ is increasing, that is, the value of θ is decreasing.

### 2.5. Simulation

**Figure 3:**
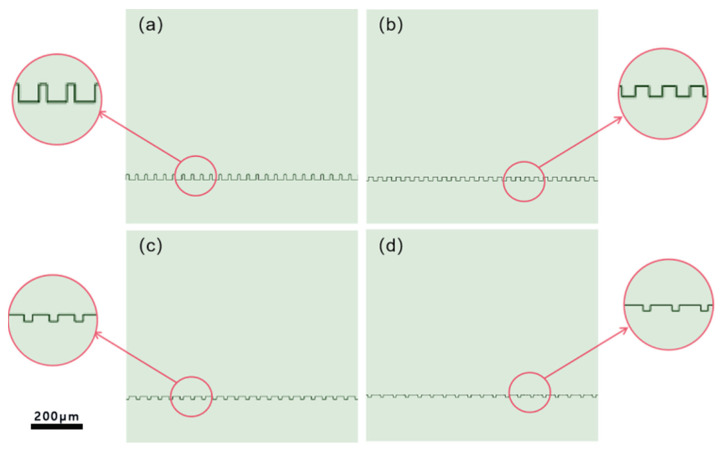
(**a**) Modeling at a T value of 5:2; (**b**) modeling at a T value of 1:1; (**c**) modeling at a T value of 1:2; and (**d**) modeling at a T value of 1:3.

To verify the relationship between square column width and contact angle, four models with varying square column widths are constructed. The notch width of the models is set to 25 μm, the square column depth is set to 20 μm, and the square column widths are set to 10, 25, 50, and 75 μm, respectively. Figure 3 depicts the four resulting models. The *T* value represents the ratio of the notch width to the width of the square column. The values for each model are 5:2, 1:1, 1:2, and 1:3, respectively.

**Figure 4:**
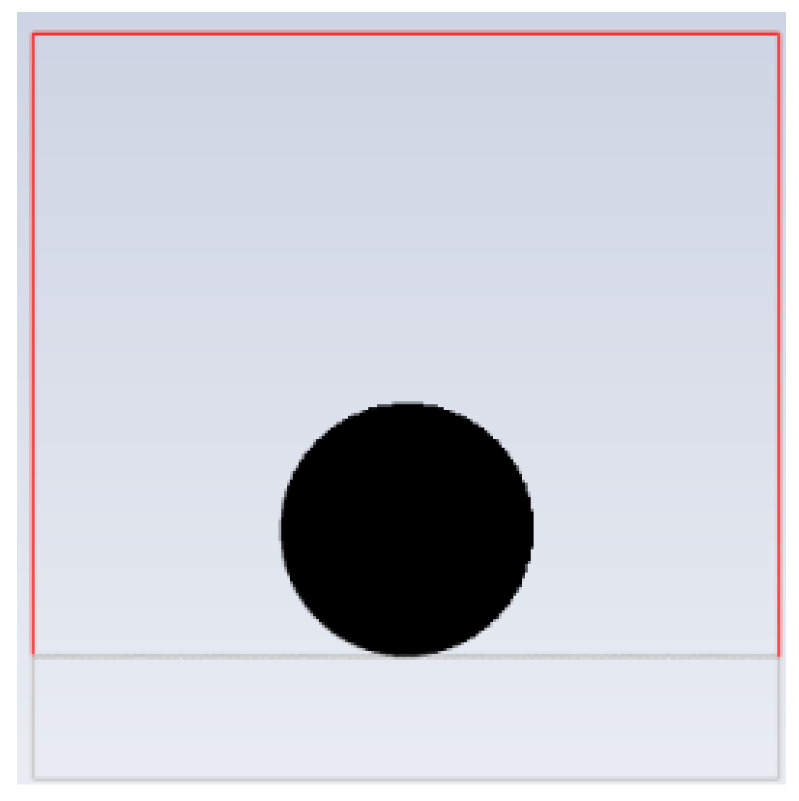
The initial state of the water droplet.

The four models were simulated in ANSYS Fluent, respectively, including grid division, parameter setting, boundary condition setting, simulation calculation, and post-processing of results. The grid unit was 0.1 mm, the gravity acceleration vertically downward was 9.8 m/s^2^, and the fluid model was the VOF (volume of fluent) module. Set the density of air to 1.225 kg/m^3^, the viscosity of air to 1.789 × 10^–5^ kg/(m∙s), the density of water to 998 kg/m^3^, and the viscosity of water to 0.001 kg/(m∙s). Set the solid material surface as a stationary no-slip wall. Draw a unit register in the fluid, initialized as a water droplet, which has a diameter of 2 mm and is initially located at a tangent to the top of the square column. Figure 4 shows the initial state of the droplet. The specific simulation steps can be seen in the Supplementary Materials.

## 3. Results and Discussion

### 3.1. Simulation Result

**Figure 5:**
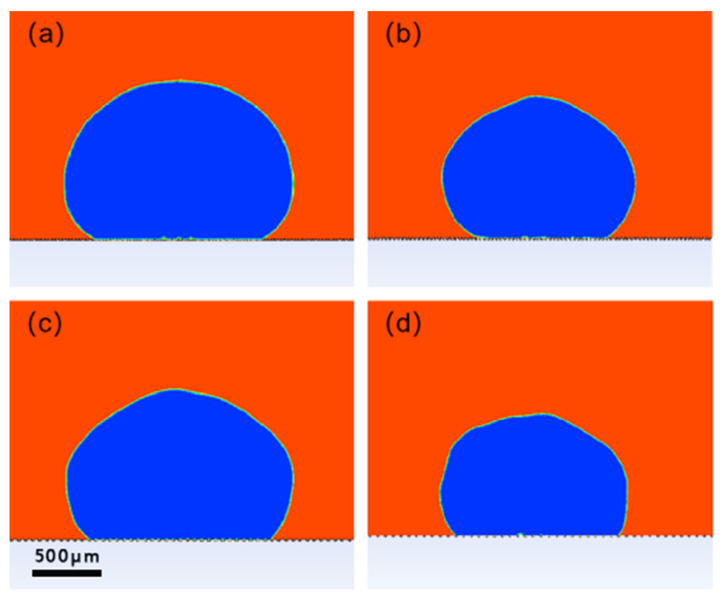
In the figure, red is the air domain, blue is the water droplets, and gray and white is the solid domain (**a**) Water droplet steady state at T value of 5:2; (**b**) water droplet steady state at T value of 1:1; (**c**) water droplet steady state at T value of 1:2; and (**d**) water droplet steady state at T value of 1:3.

The steady state of the water droplets on each rough structure was obtained at the end of the simulation and is shown in Figure 5.

The contour curve of the droplet can be obtained by analyzing and processing the simulation results with Photoshop. Subsequently, the fitted contour curves were imported into MATLAB to calculate the contact angles depicted in Figure 5a–d as 165.9°, 160.4°, 146.4°, and 146°, respectively.

The dimensions of these four structures are then utilized in the following equations for calculation. The calculated results are subsequently compared with the simulation results to validate the accuracy of the simulation.

(3) $$f_{s}=\frac{b}{a+b}(1-D\mathrm{model}),$$

(4) $$f_{s}+f_{a}=1,$$

(5) $$\cos\theta =\frac{b^{2}\cos\theta_{s}-a^{2}-2ab}{{\left(a+b\right)}^{2}}.$$

The simulation results and calculations corresponding to each value are shown in the Table 2 below.

The discrepancies between the simulation and calculated results are all below 10 percent, as presented in the table above. Therefore, we can conclude that the simulation results are consistent with the calculated results.

As the ratio of the width of the groove to the width of the square column increases, several effects are observed:

1. The contact area of the water droplets on the solid–liquid contact surface, relative to the projected area of the solid–liquid contact surface, decreases;
2. The contact angle of the water droplets on the solid surface increases;
3. The hydrophobicity of the solid surface is significantly enhanced.

### 3.2. Hydrophobic and Oleophobic Properties

**Figure 6:**
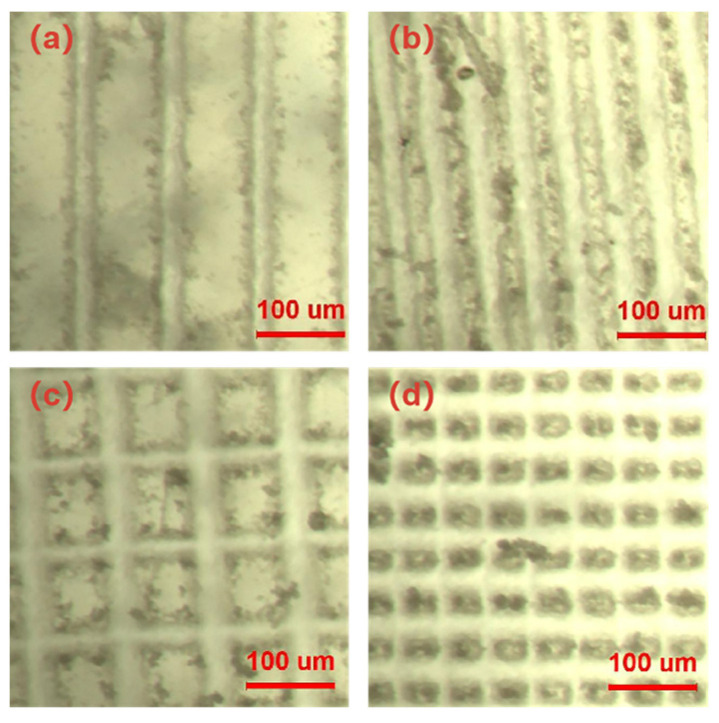
(**a**) Electron microscope images of columnar structure with a width of 75 μm; (**b**) electron microscope images of columnar structure with a width of 25 μm; (**c**) electron microscope images of grid structure with a width of 75 μm; and (**d**) electron microscope images of grid structure with a width of 25 μm.

**Figure 7:**
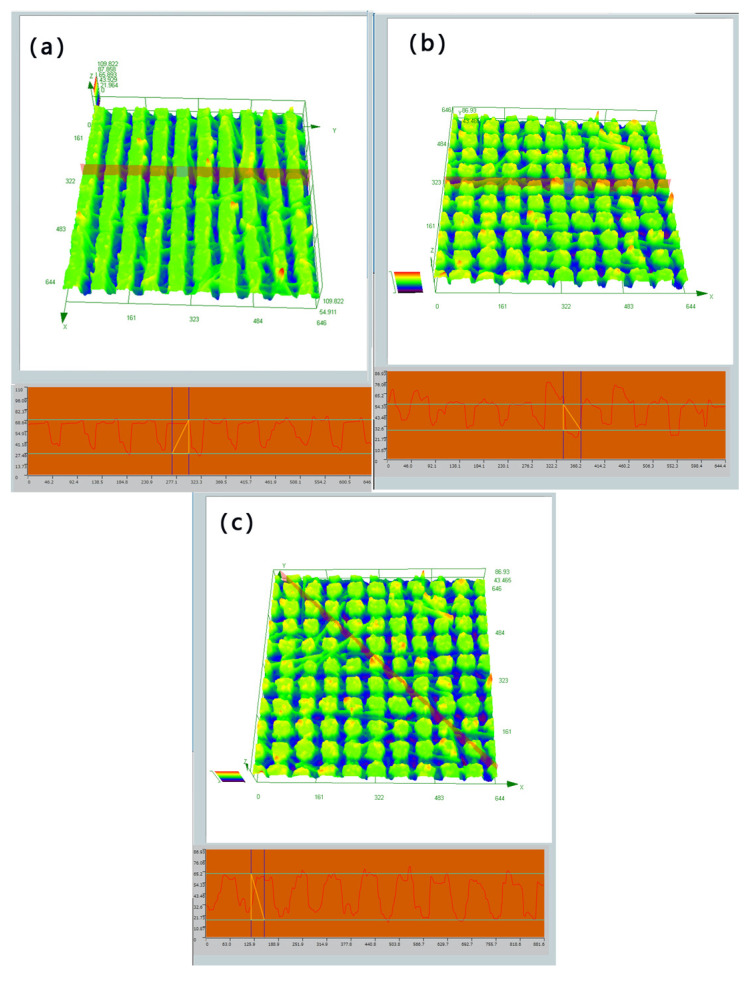
The green part in the picture is the scanned surface structure, and the red curve at the bottom is the contour curve of the section selected in the figure (**a**) Microstructure and cross-sectional profile of the columnar surface under a confocal microscope; (**b**) microstructure and cross-sectional profile of the lattice surface under a confocal microscope; and (**c**) microstructure and diagonal cross-sectional profile of the lattice surface under a confocal microscope.

Four surfaces were created by varying the scanning pitch and path of the femtosecond laser, each exhibiting distinct groove spacing and structures. The rough structure of each surface can be observed below, and by employing a microscope, the raster and columnar structures can be distinguished, as shown in Figure 6. The microstructure was scanned by a confocal laser microscope, and the cross-sectional contour of the columnar microstructure was measured as shown in Figure 7a; the horizontal cross-sectional contour of the grid microstructure was measured as shown in Figure 7b; and the diagonal cross-sectional contour was measured as shown in Figure 7c. The uniformity of the machined microstructure could be directly observed by the contour curve, and it could be seen that the consistency of the columnar microstructure was poorer than that of the grid microstructure, and the width and height of the raised areas after machining were consistent. The uniformity of the microstructure was also affected by a variety of factors, such as the microscope resolution and image analysis errors.

A confocal laser microscope has both horizontal resolution (0.2–0.25 μm) and vertical resolution (0.5–0.6 μm). By controlling the horizontal and vertical movement of the carrier table, the point-by-point, surface-by-surface, and layer-by-layer scanning of the sample can be realized, and the subtle structures of different sections of the specimen can be directly observed to form a stereo image.

**Figure 8:**
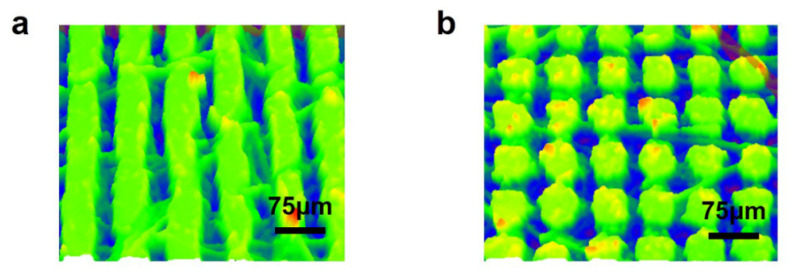
The green part is the microstructure scanned by the confocal microscope, and the blue part is the groove (**a**) Confocal microscope structure of columnar structure with a groove width of 25 μm and (**b**) confocal microscope structure of grid structure with a groove width of 25 μm.

The structure and dimensions of the machined surface were analyzed using a confocal laser microscope. The measurements revealed that the grid structure had a groove depth of 35 μm, a groove width of 25 μm, and a square column width of 25/75 μm. On the other hand, the columnar structure exhibited a groove depth of 48 μm, a groove width of 25 μm, and a square column width of 25/75 μm, as shown in Figure 8.

**Figure 9:**
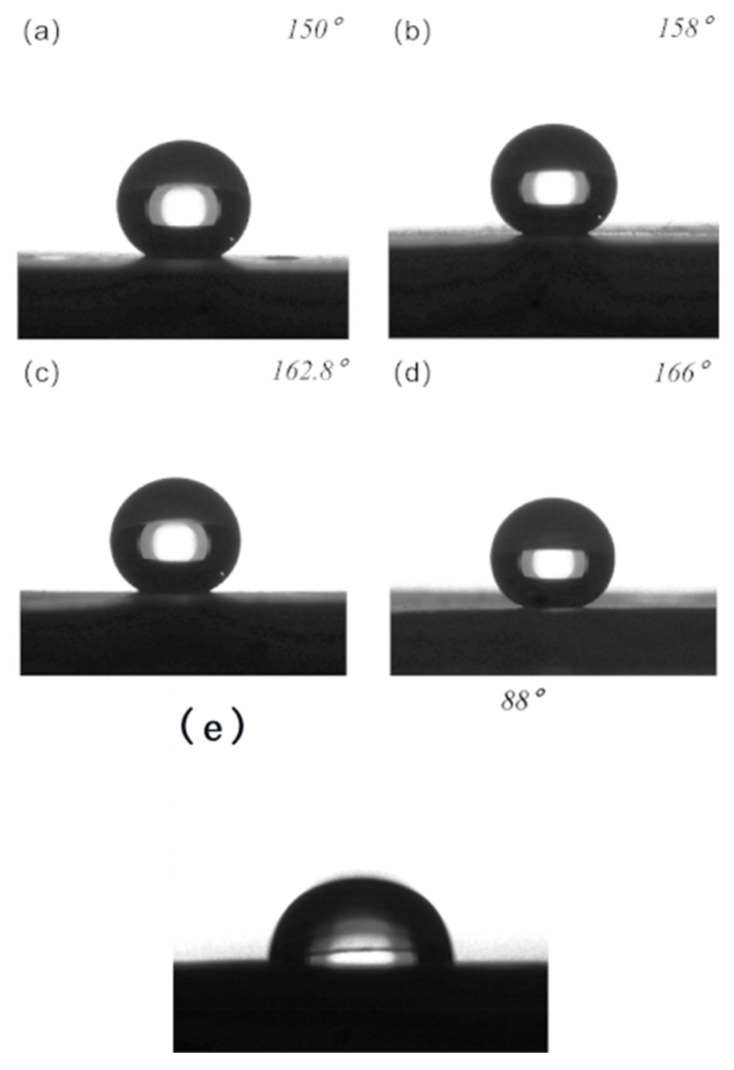
(**a**) Contact angle between the surface of a columnar structure with a square column width of 75 μm and the water; (**b**) contact angle between the surface of a column structure with a square column width of 25 μm and the water (**c**) contact angle between the surface of a grid structure with a square column width of 75 μm and the water; (**d**) contact angle between the surface of a grid structure with a square column width of 25 μm and the water; and (**e**) contact angle of water droplets on unprocessed PTFE surfaces.

The contact angle in the experiment was measured using the Kruss DSA100 contact angle measuring instrument. The contact angle of the water droplets on the surface of the unprocessed PTFE was measured to be 88°, and an image of the contact angle is shown in Figure 9e.

The contact angles of water droplets on the surfaces of the four microstructures were independently measured, yielding the following results: a contact angle of 150° was observed between water and columnar structures with a square column width of 75 μm shown in Figure 9a; a contact angle of 158° was observed between water and column structures with a square column width of 25 μm shown in Figure 9b; a contact angle of 162.8° was observed between water and grid structures with a square column width of 75 μm shown in Figure 9c; and finally, a contact angle of 166° was observed between water and grid structures with a square column width of 25 μm shown in Figure 9d.

A superhydrophobic surface has a contact angle greater than 150° and a slide angle less than 10°. According to the measurements presented previously, each structure possessed a high contact angle and exhibited excellent hydrophobicity. Currently, the slide angle of each surface is being measured to determine the effect of the micro–nano system on the slide angle of the character.

Experiments show that the PTFE surface has a solid adhesion to water; even when the sheet is inverted, water drops do not fall off the surface, so the rolling angle of water drops on the PTFE surface is defined as 90°.

**Figure 10:**
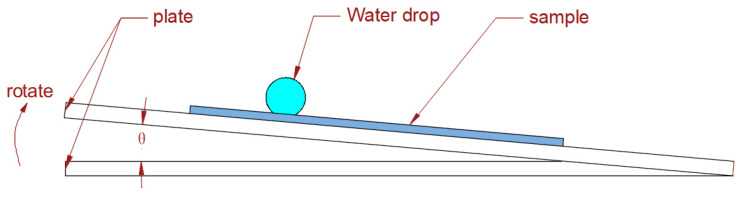
Schematic diagram of the sliding angle experiment; θ is the angle of inclination of the plate.

**Figure 11:**
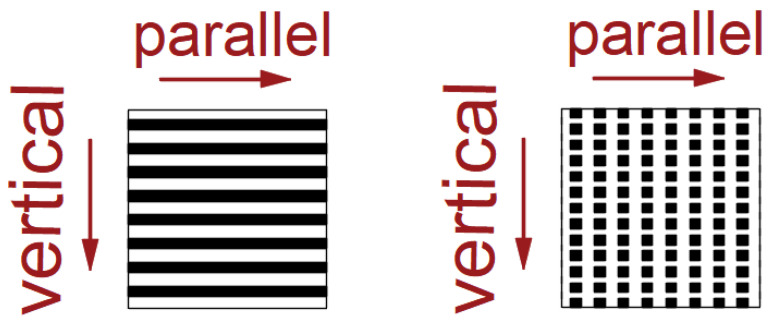
Schematic diagram of droplet sliding direction on the sample.

**Figure 12:**
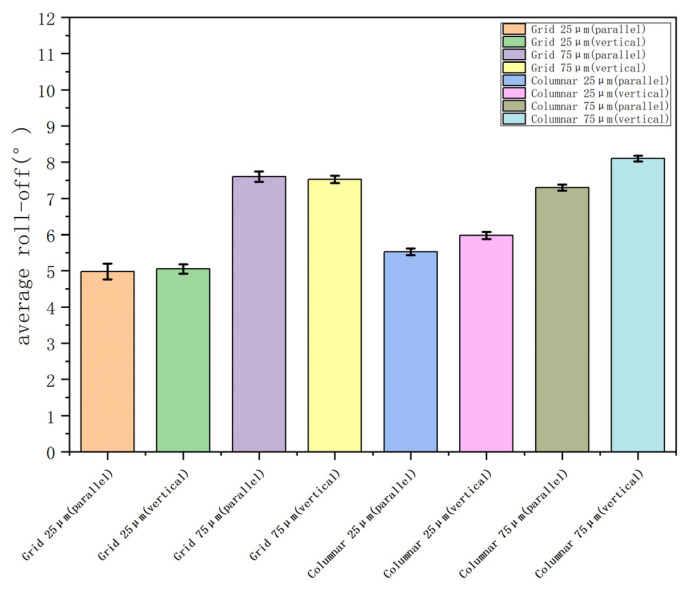
Average roll-off angles of different size structures.

The investigation of roll-off angles is crucial for determining the sliding behavior of droplets on superhydrophobic surfaces, and the measurement of roll-off angles is conducted. The experimental procedure encompasses the following steps: First, a thorough cleaning of the PTFE sample is performed to ensure its purity. Subsequently, a static contact angle is measured as a reference point. Then the sample was fixed on the plate, and 7 μL of water droplets were dripped on the sample surface. The plate angle was slowly increased from 0°. When the water droplets rolled off the sample, the increase in plate angle θ was recorded. The experimental schematic diagram is shown in Figure 10. The sliding angles of each sample structure were measured in two directions: the vertical direction of droplet sliding and the horizontal direction of femtosecond laser processing. As shown in Figure 11, the sample was schematically designed, with black indicating the bulge after processing and white indicating the groove after processing. The roll-off angles obtained by repeated experiments are shown in Figure 12.

Through the data after the experiment, it was found that the roll-off angle of the same size is the same in the horizontal and vertical directions on the grid structure, and the roll-off angle of the same size is smaller in the horizontal direction than in the vertical direction on the columnar structure.

**Figure 13:**
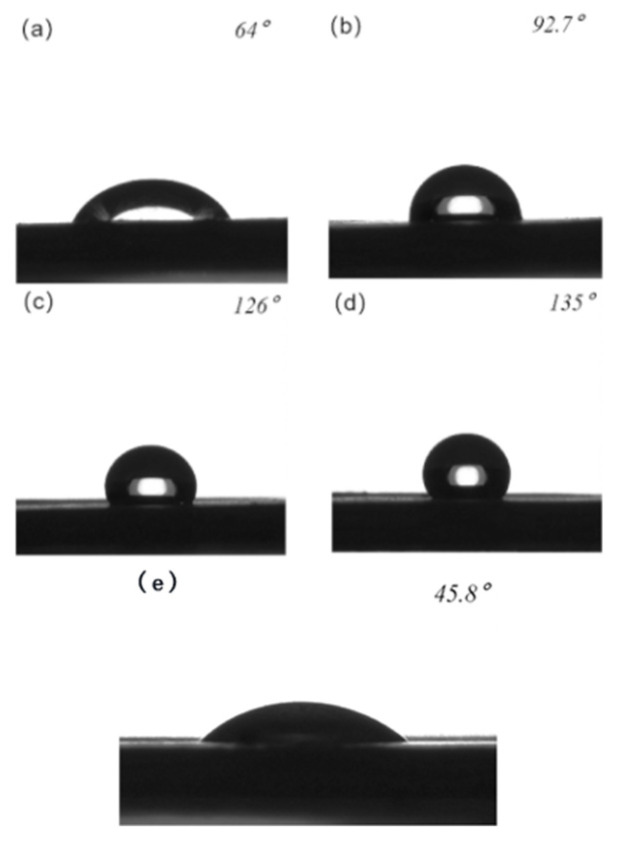
(**a**) Contact angle between the surface of a columnar structure with a square column width of 75 μm and the oil; (**b**) contact angle between the surface of a column structure with a square column width of 25 μm and the oil (**c**) contact angle between the surface of a grid structure with a square column width of 75 μm and the oil; (**d**) contact angle between the surface of a grid structure with a square column width of 25 μm and the oil; and (**e**) contact angle of unprocessed PTFE surface oil.

The contact angle of 4 µL of sunflower oil commonly found in supermarket droplets on an untreated PTFE sheet was then measured using contact angle gauges. The contact angle of the oil droplet on the surface of the untreated PTFE was estimated to be 45.8°, as shown in Figure 13e.

The contact angles of oil droplets on the surfaces of the four microstructures were independently measured, yielding the following results: a contact angle of 64° was observed between oil and columnar structures with a square column width of 75 μm; a contact angle of 92.5° was observed between oil and column structures with a square column width of 25 μm; a contact angle of 126° was observed between oil and grid structures with a square column width of 75 μm; and finally, a contact angle of 135° was observed between oil and grid structures with a square column width of 25 μm. Images depicting these measurements are shown in Figure 13.

Combining the previously derived values, the experimental results agreed with the inference, corresponding to the contact angle with the water droplet corresponding to each surface value, as shown below. The f_s_ value of the 2-D structure is calculated by Formula (6), and the results are shown in Table 3.

(6) $$f_{s}=\frac{b^{2}}{{\left(a+b\right)}^{2}}(2-D\;\mathrm{model})$$

The experimental results demonstrate a significant disparity in the contact angle between water and oil on superhydrophobic surfaces compared to unprocessed surfaces, primarily due to the strong interaction forces between water and solid surfaces. On superhydrophobic surfaces, a coating or a micro/nanostructure is applied, resulting in the formation of tiny air holes or protrusions at specific angles. These features effectively separate water molecules from the surface, thereby reducing the contact area and interaction forces between water molecules and the surface, leading to an increased contact angle for water. Conversely, oil molecules are larger than water molecules and tend to form stable molecular layers on surfaces, resulting in reduced interactions with the surface. Consequently, there is only a marginal difference observed in the contact angle of oil between superhydrophobic and ordinary surfaces.

## 4. Conclusions

This paper introduces existing natural and artificially created superhydrophobic surfaces and provides examples of their applications in relevant fields. It focuses on preparing different surface microstructures on PTFE surfaces using femtosecond laser processing technology. Femtosecond laser processing is simple and can process a wide range of materials with sub-micron accuracy.

The effects of varying groove sizes and shapes on the hydrophobicity of the PTFE surface are investigated. The water droplet states on various micro–nanostructured surfaces are simulated using ANSYS 2021 software, and the data is fitted using MATLAB 2021a to calculate the contact angles of each character. The simulation results help design more precisely sized microstructures for femtosecond laser processing. The main achievements of this study are as follows:

1. Combining theoretical derivation based on the Cassie model with experimental data, the study concludes that for PTFE surfaces with different microstructure patterns, the larger the percentage of droplet–solid contact area relative to the projection area of the solid–liquid contact interface, the higher the contact angle and the better the superhydrophilicity of the surface;
2. The study uses ANSYS software to simulate droplet states on various microstructure surfaces, guiding the design of the dimensions for femtosecond laser processing. The simulation results are in good agreement with the experimental results. The study successfully applies femtosecond laser processing technology to micro–nanostructuring the PTFE surface, producing a superhydrophobic surface with a contact angle of 166° and an oil contact angle of 135°.

Undoubtedly, there is still much work to be undertaken in this field. This study’s microstructure patterns generated through femtosecond laser processing were relatively simple, and the range of processed sizes was limited. More experiments are needed to determine the optimal superhydrophobic dimensions for specific microstructure patterns. Additionally, beyond PTFE, it is worth exploring the use of other materials. The approach of femtosecond laser processing for microstructuring surfaces is being increasingly adopted due to its unique processing capabilities and holds great promise for future applications.

## Figures and Tables

**Table 1:** Femtosecond laser processing parameters.

Processing Parameters	Laser Fluence	Scanning Speed	Repetition Rate	Pulse Duration	Scanning Pitch	Scanning Route
Grid	1.43 J/cm^2^	500 mm/s	500 kHz	500 fs	50/100 μm	Lines
Columnar	1.43 J/cm^2^	500 mm/s	500 kHz	500 fs	50/100 μm	Crossed

**Table 2:** Simulation results and calculation results corresponding to each T Value.

*T*	1:3	1:2	1:1	5:2
Simulation Results	146°	146.4°	160.4°	165.9°
Calculation Results	132°	137.4°	148.4°	162.1°
Error Value	9.6%	6.1%	7.5%	2.3%
$f_{s}$	0.75	0.67	0.5	0.29

**Table 3:** Value of $f_{s}$, contact angle, oil contact angle, and slide angle for each structure.

Structure	Columnar 75 μm	Columnar 25 μm	Grid 75 μm	Grid 25 μm
$f_{s}$	0.75	0.5	0.56	0.25
Contact angle	150°	158°	162.8°	166°
Oil contact angle	64°	92.7°	126°	135°

## Data Availability

The data supporting the findings of this study are available within the article and its Supplementary Materials.

## Supplementary Materials

The following supporting information can be downloaded at: https://www.mdpi.com/article/10.3390/polym16010043/s1.
