# Supplementary material for: Preparation of Polytetrafluoroethylene Superhydrophobic Materials by Femtosecond Laser Processing Technology
Source: Polymers (Basel). 2023 Dec 21;16(1):43. doi: 10.3390/polym16010043 (PMC10780796; doi:10.3390/polym16010043)
Supplement: Supplementary file 1 [file polymers-16-00043-s001.zip › polymers-2703452-supplementary.pdf]

1.Building 2D Models

The calculation area is set to be 6 mm×6 mm, where the solid area is 1 mm×6 mm and the watershed is 5 mm×6 mm. Set the width of the notch to be a, the depth of the square column to be h, and the width of the square column to be b.

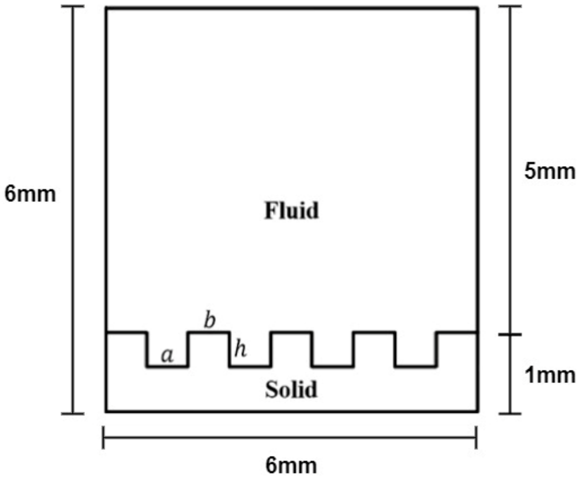

Figure S1 Schematic of the 2D model

Set a to 25 μm, h to 20 μm, b to 10、25、50、75 μm.

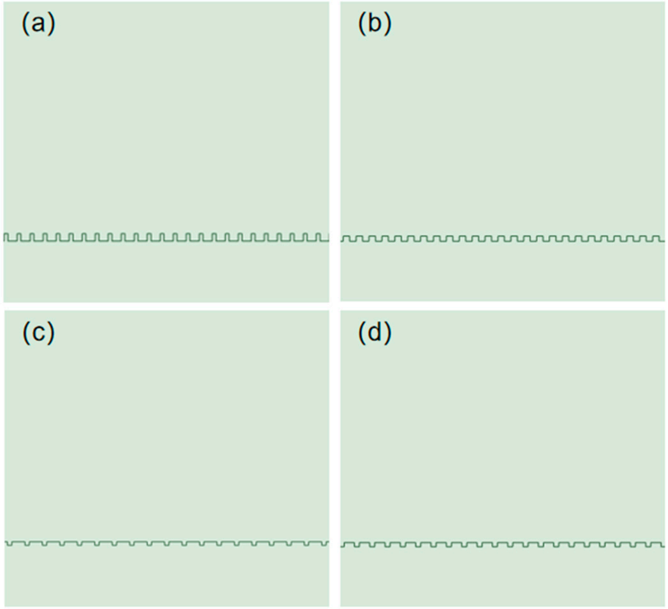

Figure S2 (a)The 2D model when b is 10 μm; (b)The 2D model when b is 25 μm; (c)The 2D model when b is 75 μm; (d)The 2D model when b is 50 μm

2.Meshing

The element size of the mesh is set to 0.1 mm and the CFD method is selected for meshing, the solver preference is selected as Fluent and the element order is selected as Linear.

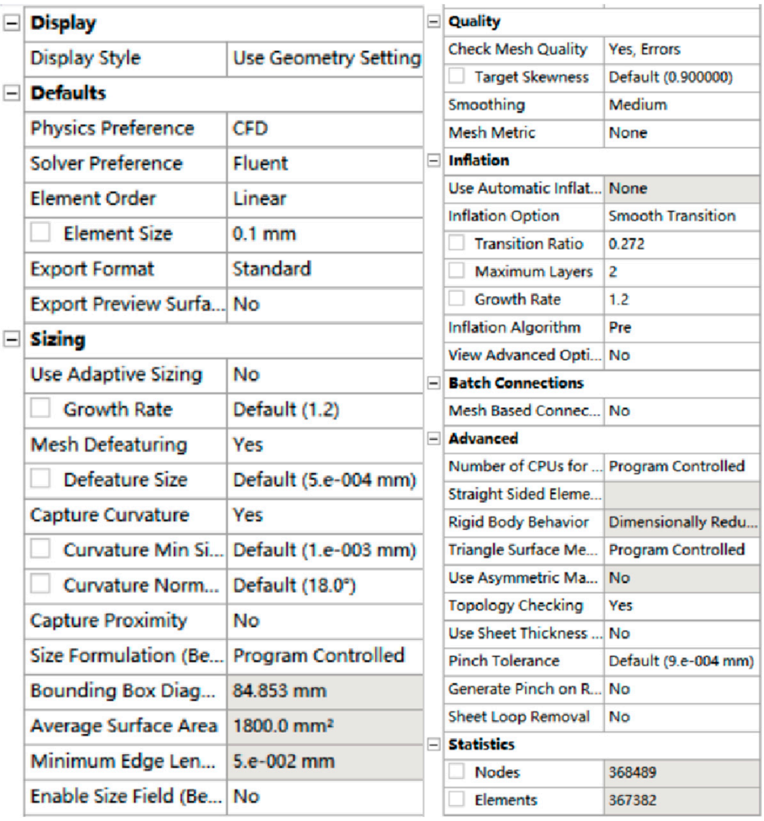

**Display**	
Display Style	Use Geometry Setting
**Defaults**	
Physics Preference	CFD
Solver Preference	Fluent
Element Order	Linear
Element Size	0.1 mm
Export Format	Standard
Export Preview Surfa...	No
**Sizing**	
Use Adaptive Sizing	No
Growth Rate	Default (1.2)
Mesh Defeaturing	Yes
Defeature Size	Default (5.e-004 mm)
Capture Curvature	Yes
Curvature Min Si...	Default (1.e-003 mm)
Curvature Norm...	Default (18.0°)
Capture Proximity	No
Size Formulation (Be...	Program Controlled
Bounding Box Diag...	84.853 mm
Average Surface Area	1800.0 mm²
Minimum Edge Len...	5.e-002 mm
Enable Size Field (Be...	No

**Quality**	
Check Mesh Quality	Yes, Errors
Target Skewness	Default (0.900000)
Smoothing	Medium
Mesh Metric	None
**Inflation**	
Use Automatic Inflat...	None
Inflation Option	Smooth Transition
Transition Ratio	0.272
Maximum Layers	2
Growth Rate	1.2
Inflation Algorithm	Pre
View Advanced Opti...	No
**Batch Connections**	
Mesh Based Connec...	No
**Advanced**	
Number of CPUs for ...	Program Controlled
Straight Sided Eleme...	
Rigid Body Behavior	Dimensionally Redu...
Triangle Surface Me...	Program Controlled
Use Asymmetric Ma...	No
Topology Checking	Yes
Use Sheet Thickness ...	No
Pinch Tolerance	Default (9.e-004 mm)
Generate Pinch on R...	No
Sheet Loop Removal	No
**Statistics**	
Nodes	368489
Elements	367382

Figure S3 Parameter Settings for Meshing

3.Models and parameterize

Set the direction of gravity to the negative direction of the y-axis with a magnitude of -9.8m/s^2.

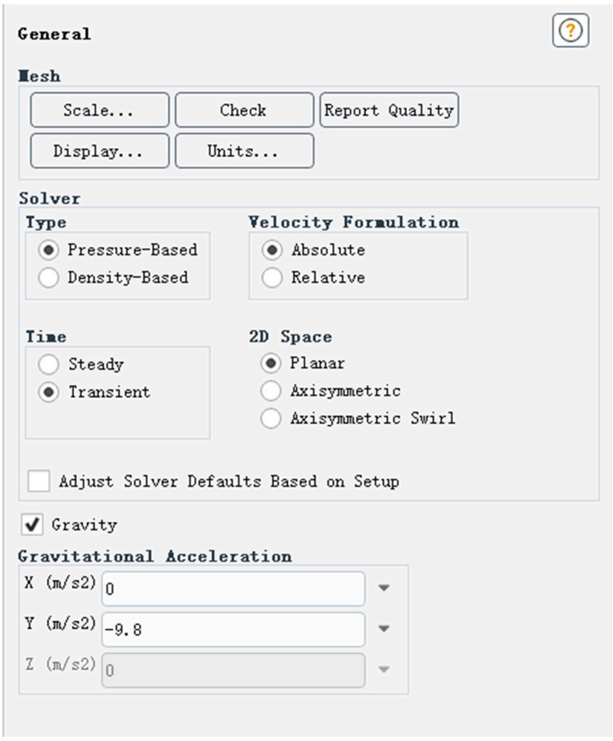

Figure S4 Gravity settings

The VOF model is selected for the simulation. Implicit body force is used in the

simulation.

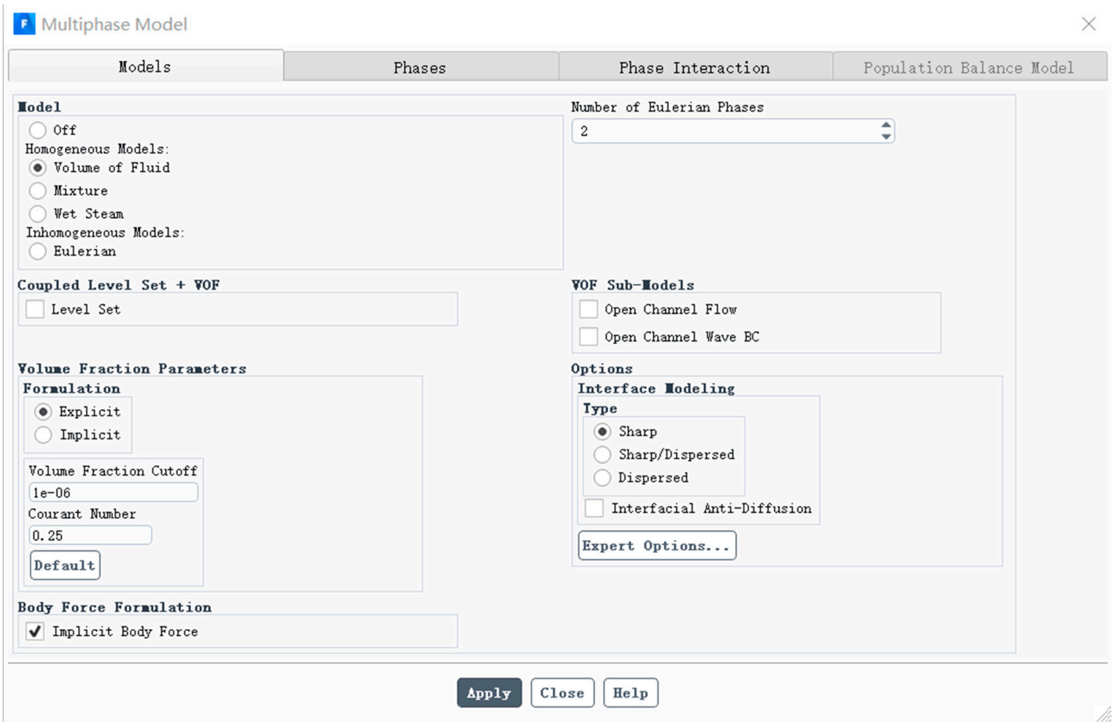

Figure S5 Model Settings

Set phase 1 to be air and phase 2 to be water.

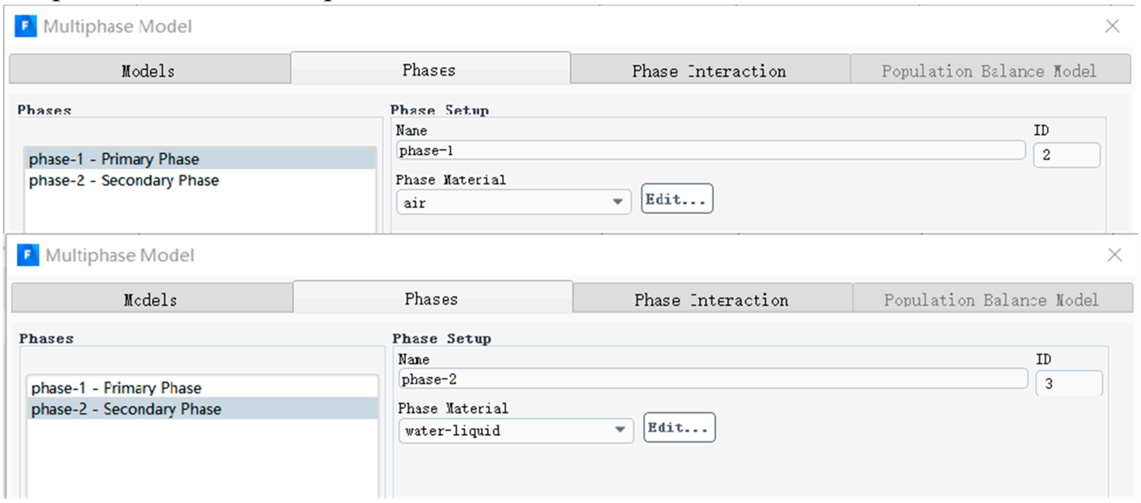

Figure S6 Phase settings

Set the density of air to 1.225 kg/m^3, the viscosity of air to 1.789×10-5 kg/(m·s) and the density of water to 998 kg/m^3, the viscosity of water to 0.001 kg/(m·s).

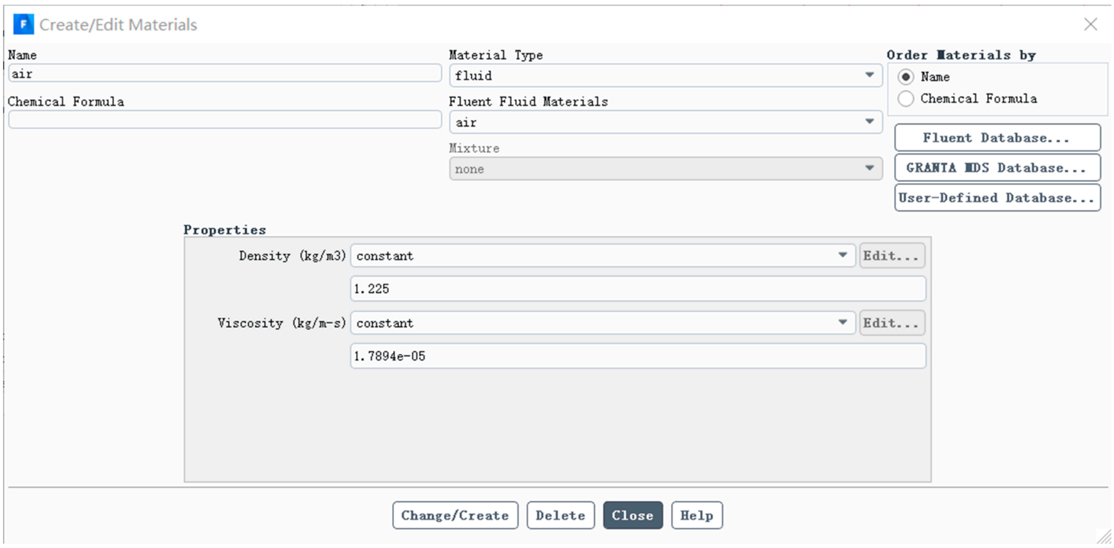

Figure S7 The parameter settings of the air

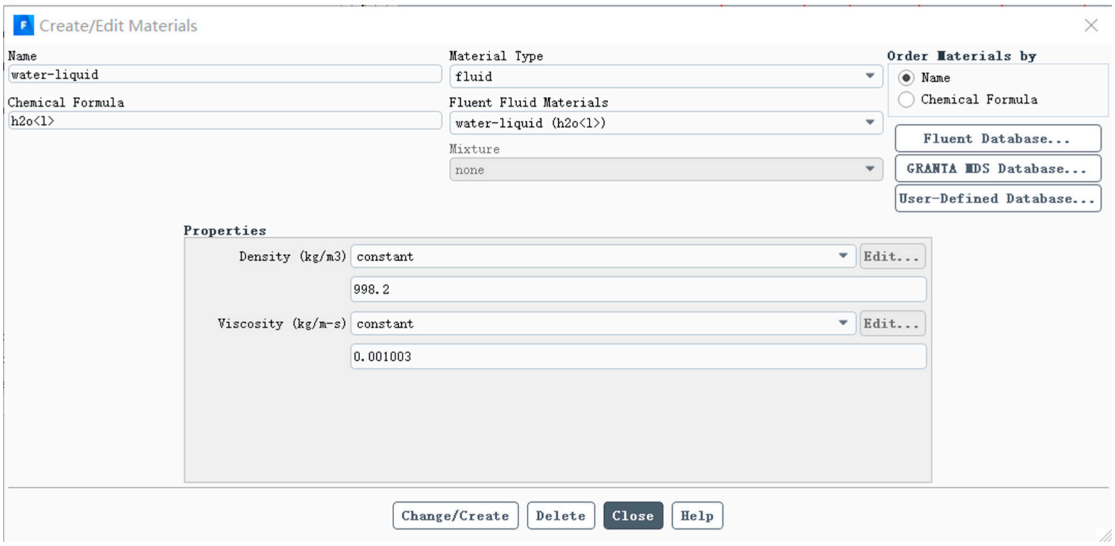

Figure S8 The parameter settings of the water

The surface tension coefficient between water and air is 0.073 N/m. Considering the effect of surface tension, surface tension force modelling is chosen and the model is selected as Continuum Surface Force and the Adhesion Options is selected as Wall Adhesion.

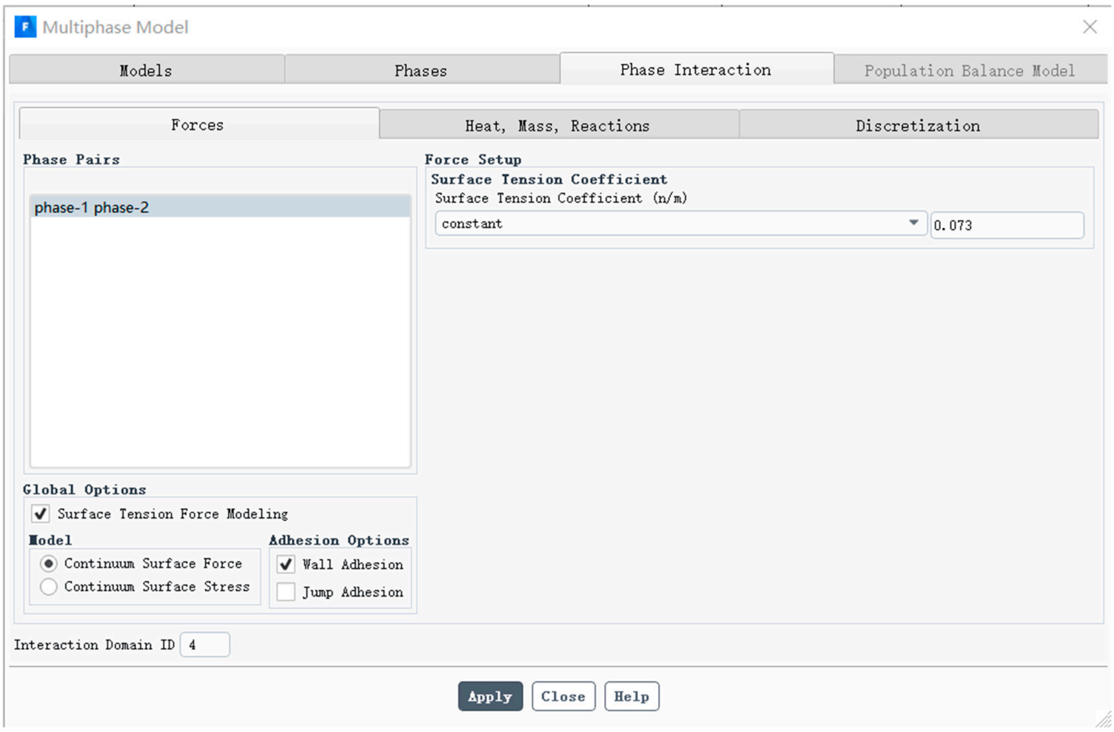

Figure S9 The settings of the phase interaction

Set the viscous model to Laminar.

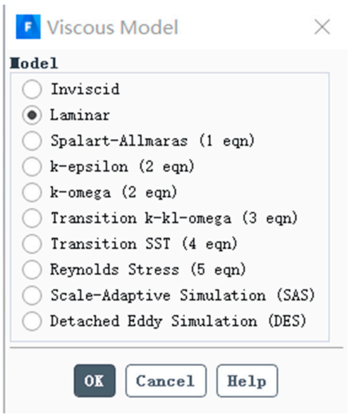

Figure S10 The settings of the viscous model

The solid material was selected as PTFE and its density was set to be 2450 kg/m^3.

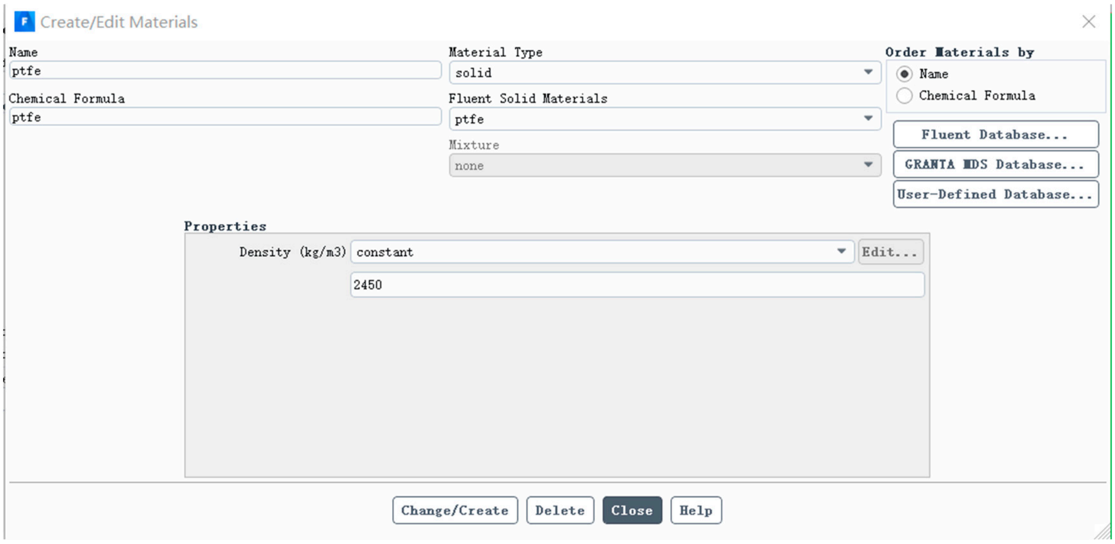

Figure S11 The parameter settings of the solid material

Set the solid material surface as a stationary No Slip Wall.

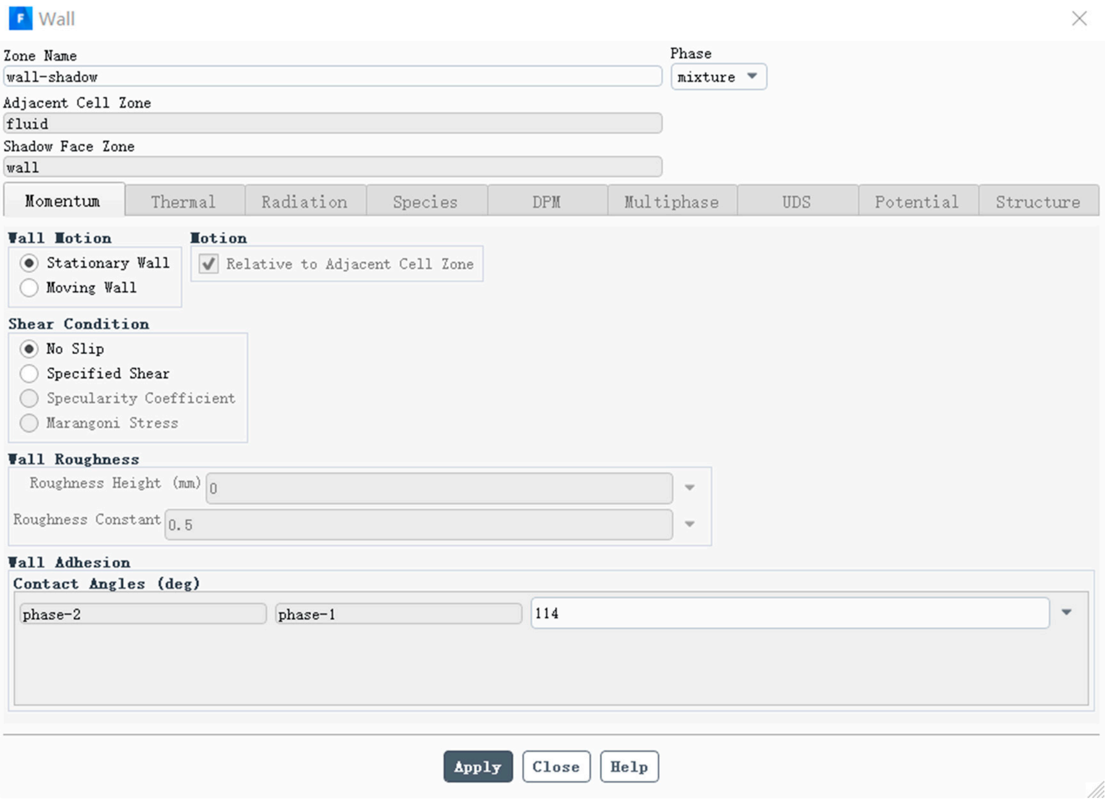

Figure S12 The settings of the solid material surface

The upper, left and right boundaries of the fluid are set as pressure outlet boundary.

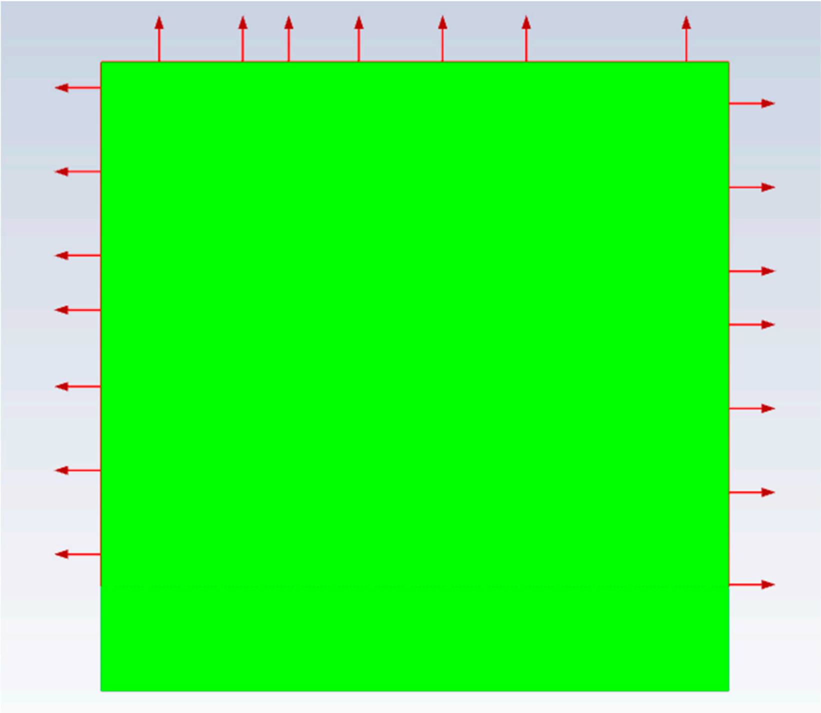

Figure S13 The settings of the pressure outlet boundary

Draw a unit register in the fluid, initialized as a water droplet, which has a diameter of 2 mm and is initially located at a tangent to the top of the square column.

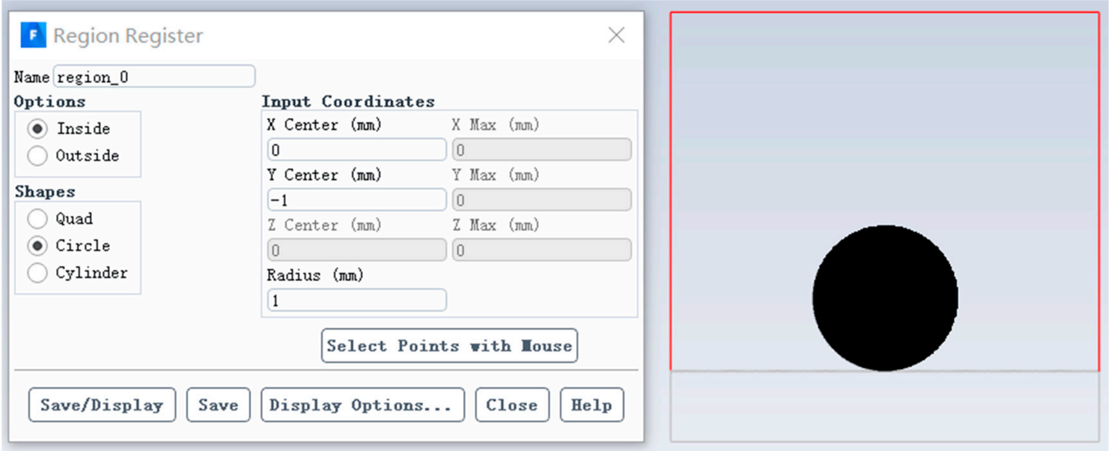

Figure S14 The settings of the droplet

The scheme is set as PISO algorithm. The gradient is Least Squares Cell Based, the pressure is Body Force Weighted, the volume fraction is Geo-Reconstruct, and the momentum is Second Order Upwind.

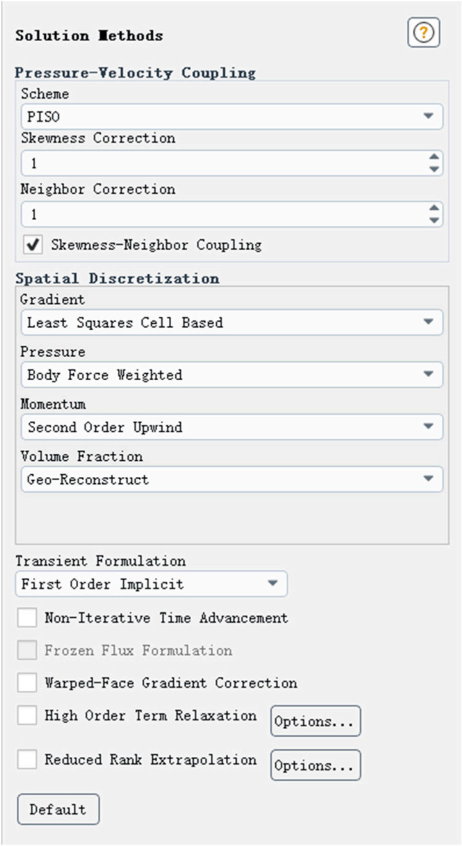

Figure S15 The settings of the solution methods

During initialization, the velocity of the droplet in both the x and y directions is set to 0, and the gauge pressure and the volume fraction are also set to 0.

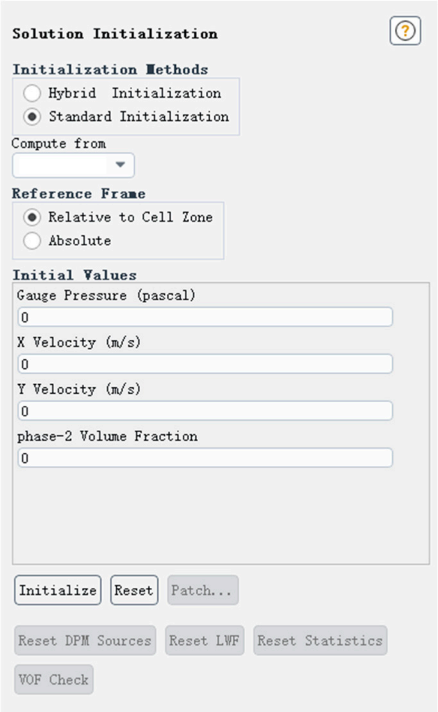

Figure S16 The settings of the solution initialization

The calculation values were set up to simulate the gradual stabilization of a water droplet on a surface over 10 ms by setting the time step size to 5e-6s, the number of time steps to 1700 and the max iteration/time step to 20.

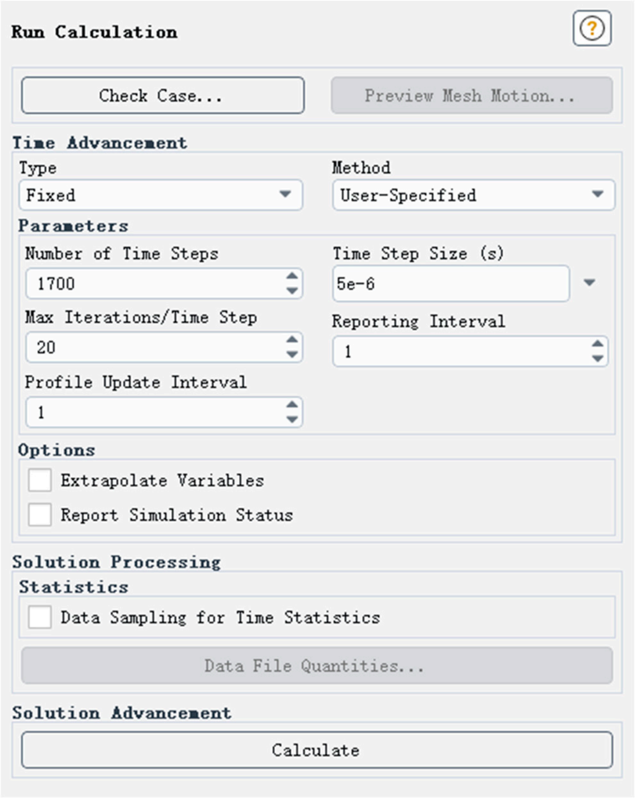

Figure S17 The settings of the calculation

Simulation Result

The steady state of the water droplets on each rough structure was obtained at the simulation's end and is shown in Fig S18.

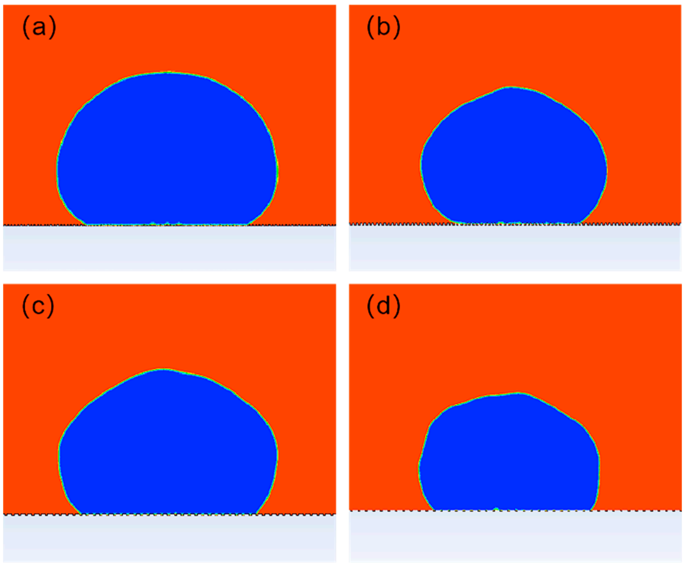

Figure S18. (a) Water droplet steady state at *T* value of 5:2; (b) Water droplet steady state at *T* value of 1:1; (c) Water droplet steady state at *T* value of 1:2; (d) Water droplet steady state at *T* value of 1:3

After obtaining the corresponding results, they were imported into Photoshop for graphic processing to enhance the droplet contours and facilitate identification by the Engauge Digitizer,as shown in Fig S19.

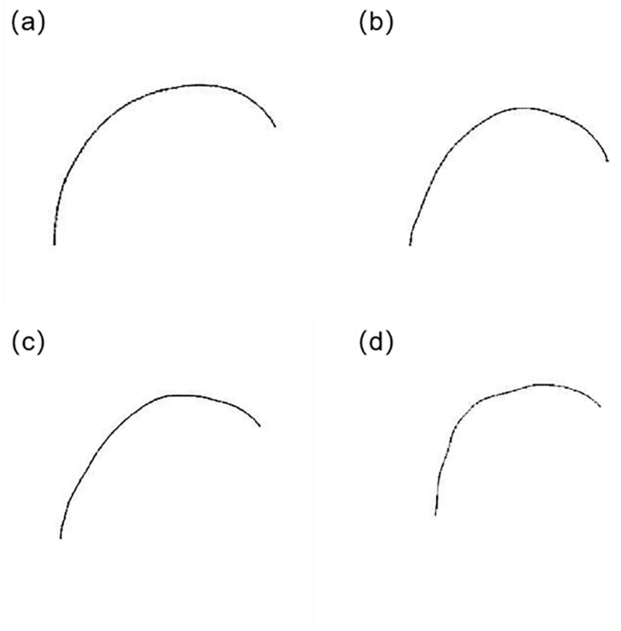

Figure S19. (a) Profile at *T* value of 5:2; (b) Profile at *T* value of 1:1; (c) Profile at *T* value of 1:2; (d) Profile at *T* Value1:3

The processed image is then imported into the Engauge Digitizer, where the aspect ratio determines the coordinate values of the three reference points, from which the values of the other coordinates on the contour line are calculated,as shown in Figure S20.

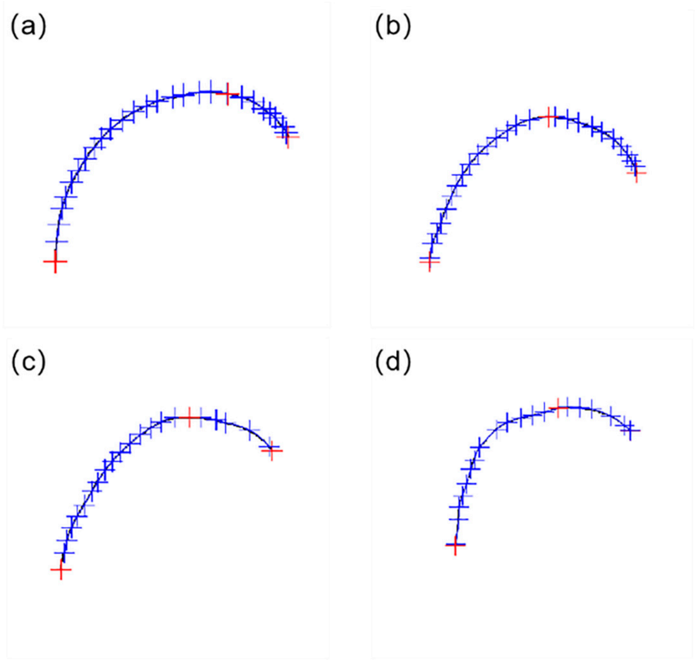

Figure S20. (a) Fitting points at *T* value of 5:2; (b) Fitting points at *T* value of 1:1; (c) Fitting points at *T* value of 1:2; (d) Fitting points at *T* value of 1:3

The coordinate values obtained from the fitting process were imported into MATLAB for curve-appropriate purposes. Each curve's equation was derived, and its corresponding value

was obtained. The contour curve fits exhibited a high level of accuracy, with a fitting accuracy of 99 percent, indicating a robust, appropriate effect. The equations of the fitted curves is shown in Figure S21.

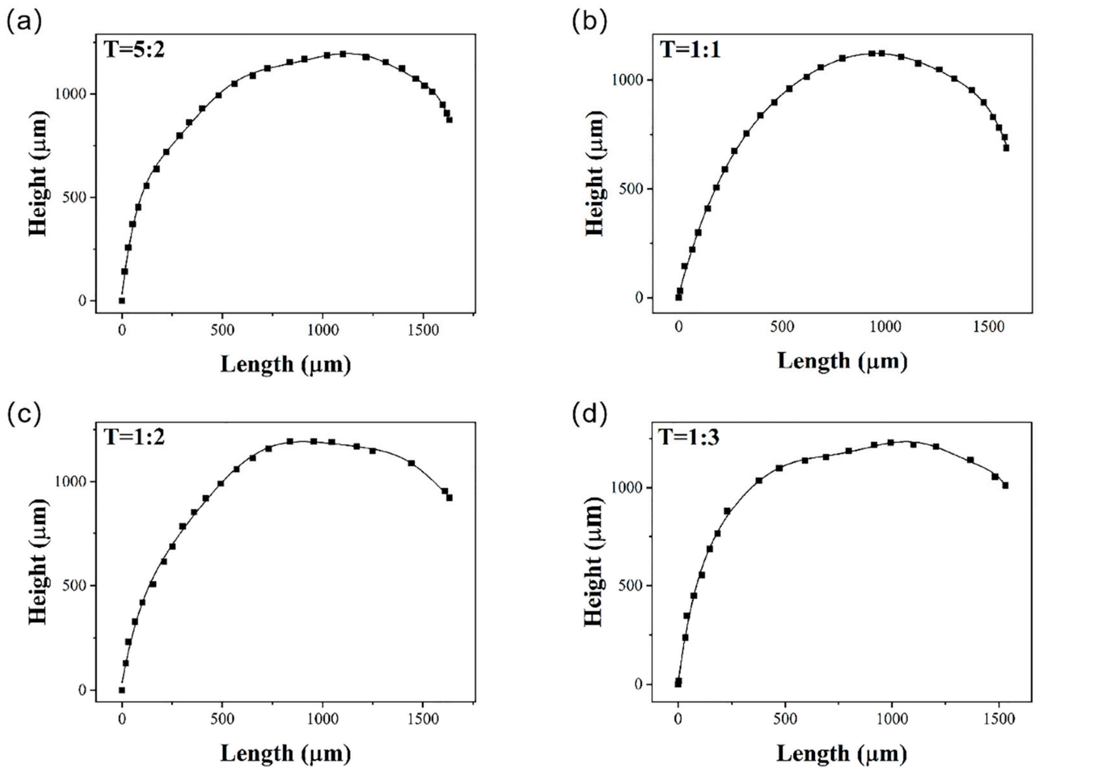

Figure S21. (a) curve of the fitted function at *T* value of 5:2; (b) curve of the fitted function at *T* value of 1:1; (c) curve of the fitted function at *T* value of 1:2; (d) curve of the fitted function at *T* value of 1:3

$T = 5:2$:

$$f(x) = -1.189\times10^{-20}x^8 + 8.176\times10^{-17}x^7 - 2.323\times10^{-13}x^6 + 3.52\times10^{-10}x^5 - 3.07\times10^{-7}x^4 + 1.556\times10^{-4}x^3 - 4.509\times10^{-2}x^2 + 8.06x + 34.04 \quad \text{(S1)}$$

$T = 1:1$:

$$f(x) = -1.237\times10^{-20}x^8 + 6.171\times10^{-17}x^7 - 1.223\times10^{-13}x^6 + 1.302\times10^{-10}x^5 - 9.852\times10^{-7}x^4 + 7.381\times10^{-4}x^3 - 5.712\times10^{-2}x^2 + 3.557x + 17.8 \quad \text{(S2)}$$

$T = 1:2$:

$$f(x) = 1.538\times10^{-18}x^7 - 1.092\times10^{-14}x^6 + 3.062\times10^{-11}x^5 - 4.363\times10^{-8}x^4 + 3.397\times10^{-5}x^3 - 1.54\times10^{-2}x^2 + 4.973x + 43.75 \quad \text{(S3)}$$

$T = 1:3$:

$$f(x) = -1.375\times10^{-20}x^8 + 8.756\times10^{-17}x^7 - 2.294\times10^{-13}x^6 + 3.199\times10^{-10}x^5 - 2.587\times10^{-7}x^4 + 1.259\times10^{-4}x^3 - 3.859\times10^{-2}x^2 + 8.279x + 9.257 \quad \text{(S4)}$$

The contact angles for each microstructure surface were determined as follows: 165.9°, 160.4°, 146.4°, and 146° based on the derived results.

The dimensions of these four structures are then utilized in the following equation for calculation. The calculated results are subsequently compared with the simulation results to validate the accuracy of the simulation.

$$f_s = \frac{b}{a+b} \tag{S5}$$

$$f_s + f_a = 1 \tag{S6}$$

$$\cos\theta = \frac{b^2 \cos\theta_s - a^2 - 2ab}{(a+b)^2} \tag{S7}$$

The simulation results and calculations corresponding to each value are shown in the table below.

Table S1. Simulation results and calculation results corresponding to each ***T*** value

T	1:3	1:2	1:1	5:2
**Simulation Results**	**146°**	**146.4°**	**160.4°**	**165.9°**
**Calculation Results**	**132°**	**137.4°**	**148.4°**	**162.1°**
**Error Value**	**9.6%**	**6.1%**	**7.5%**	**2.3%**
f_s	**0.75**	**0.67**	**0.5**	**0.29**

The discrepancies between the simulation and calculated results are all below 10 percent, as presented in the table above. Therefore, we can conclude that the simulation results are basically consistent with the calculated results

As the ratio of the width of the groove to the width of the square column increases, several effects are observed:

1、The contact area of the water droplets on the solid-liquid contact surface, relative to the projected area of the solid-liquid contact surface, decreases.

2、The contact angle of the water droplets on the solid surface increases.

3、The hydrophobicity of the solid surface is significantly enhanced.
